# The Prostate Cancer Intervention Versus Observation Trial (PIVOT) in Perspective

**DOI:** 10.4021/jocmr1395w

**Published:** 2013-06-21

**Authors:** Jian Y Cheng

**Affiliations:** Eastern Health, Department of Urology, Box Hill Hospital, 5 Arnold St, Box Hill VIC 3128, Australia. Email: Chengjy88@gmail.com

**Keywords:** Prostate Cancer Intervention Versus Observation Trial, PIVOT, Localised prostate cancer, Radical prostatectomy, Active surveillance, Watchful waiting

## Abstract

Low risk localised prostate cancer may be able to be observed rather than treated according to the Prostate Cancer Intervention Versus Observation Trial (PIVOT). This review summarises the key issues surrounding the debate in the management of localised prostate cancer, and contextualises the PIVOT’s contribution.

## Introduction

Low risk localised prostate cancer may be able to be observed rather than treated according to the Prostate Cancer Intervention Versus Observation Trial (PIVOT) [1]. Prostate cancer is now the most common cancer diagnosed in males living in developed nations. In large part this is due to the advent of Prostate Specific Antigen (PSA) screening. The PIVOT is the first well controlled trial to assess the impact of radical prostatectomy compared with observation in these patients. It finds that those with low risk disease or PSA ≤ 10 had no difference in mortality between groups, and implies that those with higher risk disease would benefit from intervention. However the issues surrounding treatment are much more complex. Treatment for prostate cancer is itself not innocuous. However observing patients carries its own risk of progression, especially because current methods of diagnosis do not distinguish aggressive from indolent tumours.

## Review

Arguably, prostate cancer is being overdiagnosed. This is largely due to the adoption of widespread PSA screening in the 1990s. This has increased the incidence of prostate cancer. It is now the most common cancer in males in developed regions such as Europe, North America and Oceania [2]. It has advanced the time of diagnosis (lead time) by 5 to 10 years [3]. Thus, more tumours are being diagnosed at an earlier stage. Data from the American prostate cancer database CaPSURE showed that those being diagnosed at stage T1 had increased 32% between 1989 and 2001, with a corresponding fall in stage T3-4 tumours [4].

As a result, there is a debate about the value of treatment in those diagnosed in the PSA age. Results are conflicting whether screening has resulted in a decrease in prostate cancer specific mortality [5, 6]. The European Randomized Study of Screening for Prostate Cancer study concluded that 48 prostate cancers needed to be treated to prevent one prostate cancer death [6]. Prior to the PIVOT, the last randomised controlled trial to study radical prostatectomy versus observation in early prostate cancer occurred prior to the widespread adoption of PSA testing [7]. This Scandinavian trial reported that radical prostatectomy decreased progression of disease, prostate cancer specific as well as all cause mortality. However these patients were more advanced than those in the PIVOT. This is further magnified given the upshifting of Gleason scores since then [4].

Moreover, in the PSA era only observational studies have compared treatment options. These include radical prostatectomy, radiotherapy or observation. One study found that 30% of those who underwent active surveillance eventually required definitive therapy in a median follow-up of 7 years [8]. Over 95% of those with low risk prostate cancer (those with Gleason score ≤ 6, PSA ≤ 10 and T stage 1 or 2a) who undergo radiotherapy and radical prostatectomy remain free of local recurrence [9].

Therefore, the PIVOT sought to identify those who would benefit from radical prostatectomy. It followed 731 men over a median of 10 years. They had an average age of 67 and a median PSA of 7.8. The participants were split into 2 arms: one with intention to treat with radical prostatectomy, the other for observation. The endpoints were all cause mortality and prostate cancer specific mortality. The men were stratified into low risk (those with Gleason score ≤ 6, PSA ≤ 10 and T stage 1 or 2a), intermediate risk (PSA 10.1 - 20, or Gleason score 7 or T2b), and high risk (PSA > 20 or Gleason score > 7 or T2c).

Its most salient result was that there was no benefit in radical prostatectomy to those with low risk disease. In those who classified as low risk patients or those with a PSA ≤ 10, radical prostatectomy did not offer any difference in rates of progression to bony metastases, prostate cancer specific or all cause mortality. This has major implications on future management of low risk prostate cancer. Currently only 10% of those eligible for active surveillance actually elect this pathway [10]. A critical reason for this may be a lack of evidence base up to this point.

Correspondingly, the PIVOT implies that those with higher risk disease who undergo surgery have gains in survival. In those with a PSA > 10, radical prostatectomy resulted in a decrease in both prostate cancer specific (5.6% vs 12.8%, P = 0.02) and all cause mortality (Hazard ratio 0.67; 95% CI 0.48 - 094). Unfortunately, its data is not definitive for these patients. Although those with intermediate risk cancer who underwent surgery had a lower all cause mortality rate, there was no difference in prostate cancer specific mortality between the groups. Conversely, those with high risk disease with surgery had less prostate cancer specific deaths but no difference in all cause mortality. This result could be due to underpowering secondary to insufficient sample size [11].

Additionally, the PIVOT’s results may not translate to younger patients with localised prostate cancer. 33% of the PIVOT’s subjects were 70 years of age or older. Unsurprisingly after a median of 10 year follow-up, roughly 50% of those enrolled had died. 85% of these deaths were due to causes other than prostate cancer. Other long term trials have found that only 5% of those with localised prostate cancer die from their disease after 20 years [10]. Therefore it is difficult to extrapolate the PIVOT’s mortality data to the 40% of prostate cancer patients who are diagnosed before the age of 65 (most with localised disease) [12]. Nevertheless, results from the PIVOT’s 15 and 20 year follow-ups would still be valuable. These can be compared with results from other large trials currently underway comparing radical prostatectomy, observation and radiotherapy.

Therefore if not quantity, then patient quality of life must factor into the decision to treat. Maintained sexual function, vitality and urinary function are associated with patient satisfaction [13]. Patients who undergo radical prostatectomy tend to have the most problems with erectile dysfunction. Almost 90% report poor erections at 2 months postoperatively. Factors affecting return of potency include patient’s premorbid state, as well as whether nerve sparing surgery was performed. Urinary incontinence is also much higher in surgical patients than radiotherapy or observation. Over 50% of patients leak urine more than once at 2 months postoperatively.

Further limiting the clinical applicability of the PIVOT is the inability to base treatment decisions on PSA levels alone. PSA currently acts as the main biochemical marker for prognosis and progression of prostate cancer [14]. However 25% of those whose disease progresses do not have a rise in PSA [15]. Thus, strategies such as measuring doubling times are dubious. Further factors affecting PSA levels include age, race, local trauma and medications. As a result management cannot be based solely on PSA.

Another challenge is the need to standardise a protocol for disease observation. Current forms consist of active surveillance or watchful waiting. Active surveillance is the postponement of curative therapy until disease advance is noted. By contrast watchful waiting focuses on symptomatic treatment in the context of progression. This avoids morbidity associated with radiotherapy and surgery. It is advantageous in those whose disease is unlikely to progress in any meaningful way. However there remains no consensus as to frequency or value of re-testing. Prostate biopsies have a high interobserver variation, and samples consist of less than 0.5% of total prostatic tissue. Therefore repeat biopsies can lead to inconsistent results [16]. Alternative markers to PSA and biopsies that are highly sensitive and specific for progression need to be developed.

The PIVOT is the latest level one evidence in an already contentious debate in the management of localised early prostate cancer. At best it shows that it is reasonable to observe low risk prostate cancer in those over 60. In these situations, the next step is to develop an optimum re-testing schedule with reliable markers of progression. In those with intermediate and high risk disease, the PIVOT suggests but cannot assert the value of radical prostatectomy over observation. Results from PIVOT at 15 and 20 years follow-up may provide further evidence. Ultimately whether or not quantity of life is increased, the patient must understand the quality of life consequences of his choice.
